# Development of an evaluation system for rational drug use in patients with chronic kidney disease using the Delphi method

**DOI:** 10.3389/fphar.2024.1183118

**Published:** 2024-10-01

**Authors:** Wenjie Yao, Xiaolan Ye, Guobing Zhang, Yan Ren, Qilong Gao, Xinfeng Ren, Yao Liu, Ping Huang, Jianlan Zheng

**Affiliations:** ^1^ Department of Pharmacy, Center for Clinical Pharmacy, Cancer Center, Zhejiang Provincial People’s Hospital (Affiliated People’s Hospital, Hangzhou Medical College), Hangzhou, Zhejiang, China; ^2^ Department of Clinical Pharmacy, Huzhou Nanxun People’s Hospital, Huzhou, Zhejiang, China; ^3^ Zhejiang Provincial People’s Hospital (Affiliated People’s Hospital, Hangzhou Medical College), Hangzhou, Zhejiang, China; ^4^ Department of Nephrology, Urology and Nephrology Center, Zhejiang Provincial People’s Hospital (Affiliated People’s Hospital, Hangzhou Medical College), Hangzhou, Zhejiang, China; ^5^ Huzhou Nanxun People’s Hospital, Huzhou, Zhejiang, China; ^6^ Department of Nursing, Urology and Nephrology Center, Zhejiang Provincial People’s Hospital (Affiliated People’s Hospital, Hangzhou Medical College), Hangzhou, Zhejiang, China

**Keywords:** medication-related problems, chronic kidney disease, Delphi technique, pharmaceutical services, healthcare, inappropriate prescribing

## Abstract

**Background:**

Chronic kidney disease (CKD) stages 3-4 present a significant clinical challenge due to the absence of a systematic approach to managing associated medication-related problems (MRPs). This lack of a structured framework hinders the timely identification and effective intervention for these complications, potentially compromising patient safety and prognosis.

**Objective:**

This study aims to leverage the Delphi method to establish an evaluation index for a rational drug use evaluation system dedicated to CKD patients in stages 3-4. This system will function as a platform for the continuous identification and management of MRPs, ultimately contributing to improved medication safety and patient outcomes.

**Methods:**

This research uses the modified Delphi technique to develop an evaluation system for rational drug use in patients with chronic kidney disease. The initial questionnaire was developed by literature review for patients with chronic kidney disease. Twenty-six senior experts formed a panel in order to evaluate items across two Delphi rounds. Consensus was defined as at least 95% agreement (first round) and 85% agreement (second round), agreeing with an average score of at least 4.5 (first round) and 4.0 (second round). Items that fulfill the stipulated criteria are eligible for inclusion in the consensus list.

**Results:**

All experts participated in both rounds (100% response rate). Consensus was achieved on three patient-related items in the first round of 34 items. Based on expert feedback, 18 revised items were included in the second round after refining, restructuring, and removing some elements. Following two rounds of consultation, 20 items achieved consensus, encompassing aspects such as drug selection, dosage assessment, treatment duration, prescription and dispensing practices, patient-related factors, and other relevant considerations.

**Conclusion:**

This study has successfully identified 20 key evaluation indicators for a rational drug use evaluation system specifically designed for CKD patients in stages 3 and 4. This system will serve as a tool for continuous MRP identification and timely intervention, ultimately enhancing medication safety and patient prognosis.

## 1 Introduction

A growing number of people are suffering from chronic kidney disease (CKD), which has becoming a global health concern (Hill et al., 2016). Chronic kidney disease is defined by either an estimated glomerular filtration rate (eGFR) less than 60 mL/min/1.73 m^2^ for 3 months or more or the presence of kidney damage, regardless of eGFR. Recently, research has indicated that 1.07% of Chinese adults had CKD, with a prevalence rate of 1.20%, 0.04% and 0.02% for CKD stages 3, 4, and 5, respectively (Zhuang et al., 2022). CKD stages 3-4 are critical periods for treatment and delaying the progressive deterioration of renal function. Timely intervention, including improved prescribing methods and medication usage, is crucial to slow the progression (Cardone et al., 2010). However, patients with CKD are medically complex, and the prevalence of medication-related problems (MRPs) increases as the disease progresses and additional medications are used. This is due to their unique physiology, related comorbidities, and the complex medication regimens comprised of several necessary medications (Mason and Bakus, 2010; Mason, 2011; Yang et al., 2016). Medication-related problems (MRPs) refer to events or circumstances pertaining to drug therapy that have the potential to either actually or potentially impede the achievement of desired health outcomes (Pharmaceutical Care Network Europe Association, 2020). In general, MRPs are generally associated with the risk of kidney injury and may contribute to the progression of CKD (Cardone et al., 2010; Fink and Chertow, 2009).

MRPs are common in all stages of CKD (Cardone et al., 2010). On the one hand, renal impairment significantly affects the pharmacokinetics of drugs, especially absorption and excretion (Eyler and Shvets, 2019; Roberts et al., 2018). Drug absorption may increase due to an impaired or decreased intestinal barrier function and/or expression of outflow transporters (such as p-glycoprotein), while drug excretion often decreases in CKD patients due to the reduced efficiency of biotransformation (Yang et al., 2016; Velenosi and Urquhart, 2014). Consequently, this leads to increased plasma levels of drugs and a greater risk of toxicity. Therefore, there is a risk of MRPs due to the changes in drug exposure and potential adverse outcomes. Second, with CKD progression, patients often develop concurrent conditions that require the intervention of several drugs at the same time (Tieu et al., 2016). The average number of medications prescribed to CKD stage 3 and 4 patients is around 6-8, while 10 to 12 different medications are prescribed to those of CKD stage 5 (Cardone et al., 2010; Mason, 2011). Studies have shown a high prevalence (93%–100%) of MRPs in CKD patients (Njeri et al., 2018), with differences between CKD stage 3 and 4 despite similar medication usage. It has been reported that the possibility of MRPs caused by inappropriate drug selection and drug overdose in patients with stage 4 CKD were 5.9 and 4.7 times that in patients with stage 3 CKD, respectively (Njeri et al., 2018). MRPs are associated with significant morbidity, leading to preventable hospitalizations and interfering with expected health outcomes, ultimately increasing the economic burden on patients (Cardone et al., 2010; Njeri et al., 2018). Evidence suggests that pharmacist interventions can improve CKD patient outcomes (Salgado et al., 2013; Castelino et al., 2011), highlighting the importance of continually identifying and addressing MRPs. While independent review by clinical pharmacists is ideal (Jung-Poppe et al., 2022), limited personnel resources often constrain their participation in detecting MRPs. Therefore, establishing a rational drug use evaluation system for CKD patients may be a feasible and effective measure to address this issue. The established evaluation system for rational drug use is a comprehensive evaluation system, designed to ensure that the scientific integrity, safety, and economic efficiency of drug administration. It mainly focuses on the selection of drugs, the method of use, the dose, the course of treatment, and the matching degree of drugs and patients’ conditions, so as to achieve the best drug treatment effect and reduce unnecessary medical risks and costs. A diverse range of tools and systems exist that facilitate doctors and patients in achieving enhanced, rational drug utilization and optimization, thereby augmenting treatment efficacy, minimizing drug-related side effects, and eliminating waste. These tools or systems may include electronic prescribing systems, drug information databases, clinical decision support systems, etc (Reeve, 2020).

The Delphi technique is a widely used group judgment method in various disciplines, including pharmacy-related studies, that seeks expert consensus through structured iteration. This allows participants to adjust their initial responses and facilitators to effectively compile the information (Diamond et al., 2014; Drumm et al., 2022). In this study, we employed the Delphi method to establish a consensus on rational drug use evaluation for application in pharmaceutical care. This system aims to identify potential medication-related problems in advance, thereby reducing their occurrence.

## 2 Methods

This study utilized the Delphi technique and adhered to the Guidance on Conducting and Reporting Delphi Studies (CREDES) (Jünger et al., 2017). As an important international documents, the Helsinki Declaration clearly define the ethical principles and restrictions that should be followed in biomedical research using human beings as subjects (World Medical Association, 2008; Emanuel, 2013). In the context of this research, ethical approval is not a prerequisite for the consensus-based list, as the study did not encompass human subjects or involve human research.

The indicators for the evaluation system of rational drug use in CKD were determined through two consecutive rounds of a Delphi survey. Both rounds were completed electronically using anonymous questionnaires. Data collection for the first survey occurred from August 25th to 1 September 2022, followed by the second survey from October 28th to 4 November 2022.

### 2.1 Literature review

We searched multiple databases, including PubMed, Web of Science, China National Knowledge Infrastructure (CNKI), and some guideline search websites like the National Institute for Health and Care Excellence (NICE), and the Kidney Disease Improving Global Outcomes (KDIGO) for English and Chinese literature published before August 2022. The full search terms used included: “Delphi,” “pharmaceutical care,” “pharmaceutical services,” “rational drug use,” “pharmaceutical administration,” “drug related problems,” “medication-related problems,” “chronic kidney disease,” “clinical pharmacokinetics,” “polypharmacy,” “medication errors,” and “inappropriate prescription”.

### 2.2 Expert panel selection

The members of the Delphi panel must have an in-depth knowledge of the issues under study and diverse perspectives, as well as a high degree of credibility in the scientific field related to kidney disease. Drawing upon an exhaustive literature review and combining with the information of relevant personnel involved in the issues, we determined the number of participants and formed an inquiry list. Subsequently, we sent formal email invitations to the members of the Delphi panel, and comprehensively considered the feedback from each expert, that is about whether they agreed to participate in this study. After comprehensive evaluation, we finally determined the list of experts participating in this study. Finally, the Delphi study panel consisted of 26 senior experts from different fields, including nephrologists, nephrology nurses and clinical pharmacists experienced in caring for patients with CKD, health policy and management experts, health economists and medical insurance specialists meeting the requirements of a Delphi study. Here are the selection criteria for all experts: 1) a bachelor’s degree or higher; 2) at least intermediate English proficiency; 3) at least 5 years of working experience; and 4) ensure the completion of two rounds of questionnaire.

### 2.3 The initial questionnaire

Based on the literature review, a clinical pharmacist with an academic background in nephrology drafted the initial questionnaire for the rational drug use evaluation system for CKD. The questionnaire included 8 first-level indicators and 34 second-level indicators. The first level indicators are designed to establish a distinct classification of the secondary indicators, ensuring a systematic and organized evaluation and analysis system. Panelists were requested to assess and provide comments on the secondary indicators.

### 2.4 Data collection

The first round questionnaires were emailed separately to the experts and asked for an anonymous email return, followed by a subsequent round via a professional online survey platform (www.wjx.cn). In the first round, experts were asked not to include their names and other details of their identity in the questionnaire they returned by email. Considering the complexity of filling out the questionnaire by email and the potential risk of human error, we have opted to adopt an online approach for the second round. This approach, boasting its automated data capture capabilities, is anticipated to enhance not just efficiency, but also the overall accuracy of our results. This decision was taken to mitigate any potential issues that may arise from the traditional email-based method. Through the carefully designed online questionnaire, experts only need to choose their own opinions for each item, which not only greatly facilitates the participation of experts, but also ensures the accuracy of their opinions. To maximize the response rates, each round of survey remained open for 1 week, with reminder emails sent at the beginning and end of the week (Hasson et al., 2000; Zhang et al., 2022).

### 2.5 Delphi rounds

#### 2.5.1 First round

An email, containing the study details, was sent to panelists in August 2022 for the first round questionnaire. The instructions were provided on the first page, followed by questions regarding basic information on the experts, such as gender, work experience, occupation, education, and job title. In this study, we asked experts to score the level of agreement for each indicator to be included in the rational drug use evaluation system for CKD. Panelists rated their opinions on each indicator using a 5-point Likert rating scale [strongly disagree 1), disagree 2), neutral 3), agree 4) and strongly agree 5)]. The initial practice of pharmaceutical care for CKD medication management was evaluated across 8 themes. Drug selection (theme A), dosage form (theme B), dose selection (theme C), treatment course (theme D), prescription versus modulator (theme E), drug use process (theme F), patient-related (theme G), other (theme H). Panelists were able to suggest their modifications to any themes, including wording changes, exclusions, additions, or integrations. However, to ensure the rigor of the review process and decision-making, as well as the rationality of the advice provided by the expert group members, it is imperative to formulate a clear change rationale.

After the first round, the average score, coefficient of variation and percentage of agreement (the proportion of experts who agreed and strongly agreed) were calculated. The conditions of consensus were as follows: 1) at least 95% of the panelists agreed, 2) an average score ≥4.5, 3) coefficient of variation <0.15, and 4) no other objections. Items with agreement of less than 80% or a coefficient of variation ≥0.20 were excluded. Based on expert feedback, some items were modified. Finally, items not meeting the established consensus conditions or underwent revisions based on feedback were deemed to have not attained consensus (“No”), this being included in the second round for re-rating.

#### 2.5.2 Second round

The modified questionnaire for the second round was presented to panelists who completed the first round. They were asked to re-rate items that had not reached consensus in the previous round, according to their own opinions and feedback. Items that achieved at least 85% agreement and an average score of at least 4.0 were considered to be a consensus.

### 2.6 Statistical analyses

According to published authoritative literature (Zhang et al., 2022; Lin et al., 2022), the voting results of experts are summarized, and the average score and coefficient of variation were calculated. Microsoft Office Excel 2021 and SPSS 27.0 were used to collect and analyze the data.

## 3 Results

### 3.1 Basic characteristics of the experts

There were 26 complete responses in the first round Delphi survey from 26 panelists. The gender distribution was approximately 1:3 (males:females). These panelists worked in clinical or academic fields, with work experience of 5 years or above. Over 60% (61.5%) had an experience of more than 10 years. One panelist was a director, and 9 were associate directors. All panelists held higher education degrees, 5 had a Ph.D., and 14 had a master’s degree (Table 1).

**TABLE 1:** Participants demographic characteristics.

Characteristics	Distribution	Number	Percentage (%)
Gender	Male	7	26.92
	Female	19	73.08
Work experience (years)	≤10	10	38.46
	11–15	9	34.62
	16–20	5	19.23
	>20	2	7.69
Highest level of education	Doctorate	5	19.23
	Master	14	53.85
	Bachelor	6	23.08
	Other	1	3.85
Professional title	Director	1	3.85
	Associate director	9	34.62
	Intermediate	14	53.85
	Other	2	7.69
The current area of work	Pharmacist	11	42.31
	Nephrologist	12	46.15
	Health policy and health management	1	3.85
	Health economy	1	3.85
	Medical security	1	3.85

### 3.2 Results of the Delphi rounds

#### 3.2.1 The first round

Panelists were invited to evaluate 34 items initially listed in Table 2. Three items (8.8%) reached consensus, meaning at least 95% of panelists agreed and the average score was at least 4.5. Notably, all these items belonged to theme G (Patient-related). Conversely, four items (11.8%) with an agreement below 80% and a coefficient of variation ≥0.20 were excluded. Two of these exclusions were from theme A (Drug selection), and two from theme E (Prescription and dispensing practices).

**TABLE 2:** Evaluation index of the first round.

NO	Item description	Agreement rate (%)	Average score±SD	Coefficient of variation (%)	Consensus
Theme A: Drug selection					
A1	Inappropriate drug selection (not recommended by the guidelines)	96.15	4.692 ± 0.679	0.145	NO
A2	Inappropriate drug selection (recommended by guidelines, but with other contraindications)	96.15	4.538 ± 0.582	0.128	NO
A3	Non-indicated medication	84.62	4.462 ± 0.761	0.170	NO
A4	Interaction (drug to drug, drug to herbal)	88.46	4.385 ± 0.697	0.159	NO
A5	Repeated use of drugs (same pharmacological action or same active ingredient)	92.31	4.385 ± 0.637	0.145	NO
A6	No medication, despite indications	80.77	4.269 ± 0.874	0.205	Exclude
A7	Overtreatment	76.92	4.231 ± 0.815	0.193	Exclude
Theme B: Drug dosage forms					
B1	Unreasonable drug dosage forms (for patients)	88.46	4.346 ± 0.689	0.159	NO
Theme C: Dosage assessment					
C1	Too low drug dosage	88.46	4.423 ± 0.703	0.159	NO
C2	Too high drug dosage	96.15	4.577 ± 0.578	0.126	NO
C3	Insufficient frequency of administration	84.62	4.269 ± 0.724	0.170	NO
C4	Excessive dosing frequency	92.31	4.462 ± 0.647	0.145	NO
C5	Dose timing setting is wrong, unclear or omitted	96.15	4.423 ± 0.703	0.159	NO
Theme D: Treatment duration					
D1	Too short a course	92.31	4.500 ± 0.648	0.144	NO
D2	Too long a course	96.15	4.500 ± 0.583	0.130	NO
Theme E: Prescription and dispensing practices					
E1	Unavailable drugs	76.92	4.077 ± 0.935	0.229	Exclude
E2	Necessary information not provided	76.92	4.308 ± 1.011	0.235	Exclude
E3	Wrong medication, size, and dosage (OTC) was suggested	92.31	4.731 ± 0.604	0.128	NO
E4	Dispensing the wrong medication, specification	92.31	4.731 ± 0.604	0.128	NO
Theme F: Drug use process					
F1	Improper timing or spacing of medication	100.00	4.423 ± 0.504	0.114	NO
F2	Insufficient drug dosage	96.15	4.615 ± 0.571	0.124	NO
F3	Overdosage	92.31	4.654 ± 0.629	0.135	NO
F4	Not taking medication	92.31	4.577 ± 0.758	0.166	NO
F5	Taking the wrong medication	96.15	4.615 ± 0.697	0.151	NO
Theme G: Patient-related factors					
G1	Not taking enough or no medication at all	100.00	4.808 ± 0.402	0.084	YES
G2	Over the prescribed dose	96.15	4.731 ± 0.533	0.113	YES
G3	Substance abuse (unregulated overuse)	92.31	4.654 ± 0.629	0.135	NO
G4	Unnecessary medications	80.77	4.154 ± 0.732	0.176	NO
G5	Interaction between the food and the medicine	88.46	4.308 ± 0.679	0.158	NO
G6	Improper storage of medication	88.46	4.385 ± 0.697	0.159	NO
G7	Inappropriate dosing time or interval	100.00	4.538 ± 0.508	0.112	YES
G8	The wrong use of medication	100.00	4.692 ± 0.471	0.100	NO
G9	Cannot be taken correctly as required	96.15	4.615 ± 0.571	0.124	NO
Theme H: Other relevant considerations					
H1	Efficacy monitoring is not performed or is not justified (e.g., TDM)	96.15	4.577 ± 0.578	0.126	NO

The coefficient of variation was not reached for five items in theme A (Drug selection), one in theme B (Drug dosage forms), five in theme C (Dosage assessment), two each in theme D (treatment duration) and E (Prescription and dispensing practices), five in theme F (Drug use process), six in theme G (Patient-related factors), and one in theme H (Other relevant considerations). In addition, based on expert feedback, we adjusted one item and integrated thirteen items according to expert opinions (Table 3). Experts proposed several changes: 1) moving item C5 to theme E due to overlap with existing indicators, 2) merging overlapping themes like A and B, F and G, 3) removing item G9 as a duplicate of G8, and 4) placing item H1 at the beginning for better flow. Following these suggestions and the lack of consensus, eighteen items progressed to the second round for re-evaluation.

**TABLE 3:** The revised items after the first round.

No	Items in the first round	No	Revisions
A1	Inappropriate drug selection (recommended by guidelines but with other contraindications)	A2	Inappropriate drug selection (recommended by guidelines, but with other contraindications, including intolerance)
B1	Unreasonable dosage (for patients)		
C3	Insufficient frequency of administration	C3	Inappropriate frequency of administration (insufficient or excessive)
C4	Excessive dosing frequency		
C5	Dose timing setting is wrong, unclear or omitted	C5	Medication time setting (error, unclear or omission)
F1	Improper timing or spacing of medication	G8	Cannot take medication correctly as required (dose, frequency, missed or inappropriate duration or interval of medication)
F2	Insufficient drug dosage		
F3	Overdosage		
F4	Not taking medication		
G8	The wrong use of medication		
G9	Cannot be taken correctly as required		
F5	Taking the wrong medication	G3	Overuse of medication (unregulated overuse, wrong medication, or unnecessary medication)
G3	Substance abuse (unregulated overuse)		
G4	Unnecessary medications		

#### 3.2.2 The second round

Based on the first round results, the new questionnaire was revised to include 18 items (Table 4). This revision led to seventeen (94.4%) items achieving consensus, meaning they had at least 85% agreement and an average score of at least 4.0. These items were categorized into five groups: four (22.2%) on drug selection, four (22.2%) on dosage assessment, two (11.1%) on treatment duration, two (11.1%) on prescription and dispensing practices, four (22.2%) on patient-related factors and one (5.6%) on other relevant considerations. Notably, the item regarding non-indicated medication was excluded due to panelists’ disagreement.

**TABLE 4:** Evaluation index of the second round.

NO	Item description	Agreement rate (%)	Average score±SD	Coefficient of variation (%)	Consensus
Theme a drug selection					
A1	Inappropriate drug selection (not recommended by the guidelines)	92.31	4.692 ± 0.736	0.157	YES
A2	Inappropriate drug selection (recommended by guidelines, but with other contraindications, including intolerance)	100.00	4.692 ± 0.471	0.100	YES
A3	Non-indicated medication	84.62	4.385 ± 0.941	0.215	NO
A4	Interaction (drug to drug, drug to herbal)	100.00	4.615 ± 0.496	0.107	YES
A5	Repeated use of drugs (same pharmacological action or same active ingredient)	92.31	4.538 ± 0.647	0.143	YES
Theme C Dosage assessment					
C1	Too low drug dosage	96.15	4.731 ± 0.533	0.113	YES
C2	Too high drug dosage	100.00	4.846 ± 0.368	0.076	YES
C3	Inappropriate frequency of administration (insufficient or excessive)	100.00	4.731 ± 0.452	0.096	YES
C5	Medication time setting (error, unclear or omission)	96.15	4.577 ± 0.578	0.126	YES
Theme D Treatment duration					
D1	Too short a course	100.00	4.731 ± 0.452	0.096	YES
D2	Too long a course	96.15	4.692 ± 0.549	0.117	YES
Theme E: Prescription and dispensing practices					
E3	Wrong medication, size, and dosage (OTC) was suggested	100.00	4.731 ± 0.452	0.096	YES
E4	Dispensing the wrong medication, specification	100.00	4.731 ± 0.452	0.096	YES
Theme G Patient-related factors					
G3	Receiving improper medication (utilizing incorrect and unnecessary medications, engaging in unregulated overuse)	100.00	4.731 ± 0.452	0.096	YES
G5	Interaction between the food and the medicine	88.46	4.500 ± 0.707	0.157	YES
G6	Improper storage of medication	92.31	4.654 ± 0.629	0.135	YES
G8	Cannot take medication correctly as required (dose, frequency, missed or inappropriate duration or interval of medication)	96.15	4.692 ± 0.549	0.117	YES
Theme H Other relevant considerations					
H1	Efficacy monitoring is not performed or is not justified (e.g., TDM)	100.00	4.769 ± 0.430	0.090	YES

#### 3.2.3 Delphi consultation results

**FIGURE 1:**
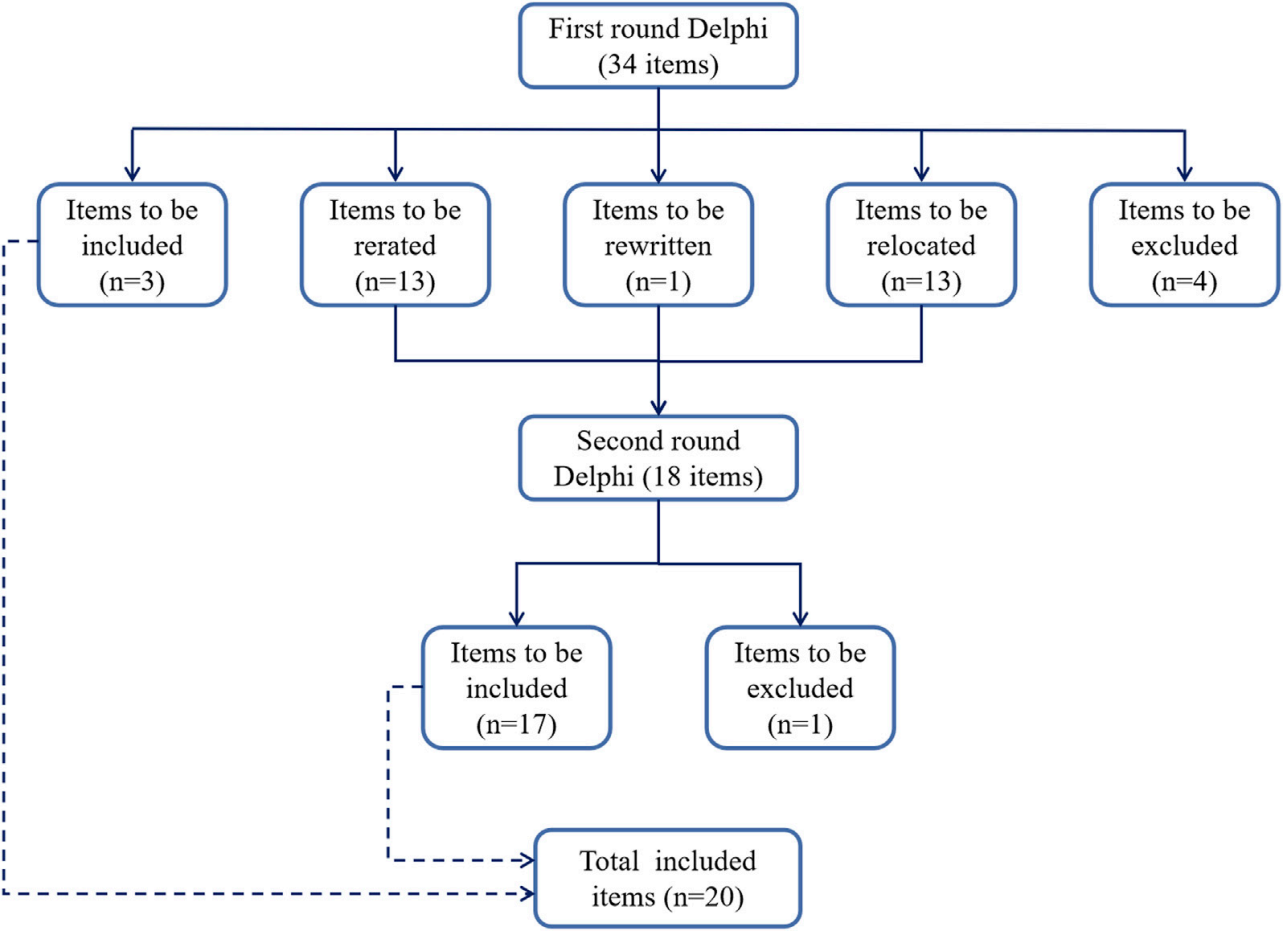
Depicts the Delphi process used in this study. Flow chart for the Delphi rounds.

The process of the Delphi study is shown in Figure 1. This study conducted two rounds of expert consultation with a perfect response rate (26/26) for both questionnaires. After the first round, consensus was reached on 3 out of 34 items. Subsequently, 4 items were excluded, 13 items were merged into 4 new items, and 1 was rewritten. The remaining 13 original and 5 modified items were carried over to the second round. Here, 1 item was excluded and 17 were considered to reach a consensus. Through this process, 20 items for the rational drug use evaluation system for CKD were initially established. Notably, consensus was reached on 3 items in the first round and 17 in the second, covering aspects like drug selection, dosage assessment, treatment duration, prescription and dispensing practices, patient-related factors and other relevant considerations.

## 4 Discussion

### 4.1 Medication-related problems

MRPs can be a source of distress for patients, negatively impacting their healthcare experience. For patients with chronic kidney disease, factors like polypharmacy (using multiple medications) and renal impairment contribute to a higher risk of MRPs, during hospitalization (Garin et al., 2021). These issues can lead to two main types of contradictions: effectiveness (not achieving desired outcomes) and adverse reactions (Garin et al., 2021). Inappropriate medication prescription is the main cause of MRPs in CKD patients. A study reported that a total of 15.18% of CKD patients experienced such prescriptions, with cautiously used medicines (9.29%), unreasonable dosage (3.23%), and contraindicated medications (2.65%) being the most common reasons. Drug and dose selection remain the key contributors to these issues (Yang et al., 2016; Garin et al., 2021). A study identified drug interactions, medication non-adherence, polypharmacy, and comorbidities, and using medications without a clear indication as the most common MRPs in CKD patients (Njeri et al., 2018). It also revealed that the incidence of MRPs was higher in patients with CKD stage 4 compared to those with stage 3.

Patients play a crucial role in successful medication management. Improving their self-management skills is vital in preventing medication-related problems such as receiving improper medication (utilizing incorrect and unnecessary medications, engaging in unregulated overuse), food-drug interactions, improper storage and incorrect dosing. For example, self-administring non-steroidal anti-inflammatory drugs (NSAIDs) can increase the risk of acute kidney injury (AKI), a well-known risk factor for chronic kidney disease progression (Baker and Perazella, 2020). Patients with CKD are particularly susceptible to AKI and repeated episodes can accelerate CKD progression (Ruiz-Ortega et al., 2020; He et al., 2017; Sato et al., 2020). Preventing and minimizing AKI events holds significant benefits for CKD patients, improving their prognosis and overall health (He et al., 2017).

### 4.2 Delphi process

The Delphi method is a systematic approach to gathering consensus from a panel of experts. It involves multiple rounds of anonymous surveys, allowing experts to express their opinions and revise them based on feedback from others. Through this iterative process, a “group judgment” emerges, representing a shared perspective on a specific issue. This method offers a highly representative and reliable way to generate insights, and it has been widely used in pharmaceutical research, particularly for developing evaluation systems.

While there is no formal requirement for the number of rounds or participants, recent studies pointing out that a point of diminishing returns is reached after exceeding rounds. Two to three rounds are generally considered optimal (Drumm et al., 2022; Hasson et al., 2000). The Delphi process continues until consensus is reached or the benefits of further rounds become negligible. The first round usually involves open-ended questions or interviews to gather initial ideas and frame subsequent rounds. By the second or third round, experts typically reach a sufficient level of agreement.

Research also suggests that 10 to 40 experts are suitable to balance the risk of bias and data analysis challenges from having too few or too many participants (Zhang et al., 2022). Our research invited 26 experienced healthcare professionals with higher education qualifications for participation, 61.5% of them possessed over 10 years of experience. Recognizing the significance of effective MRPs management in CKD patients, we strategically selected participants, with pharmacists constituting 42% and nephrologists comprising 46% of the specialists (Diamond et al., 2014).

### 4.3 Strengths and limitations of the study

The Delphi technique, well-suited for gathering expert consensus, was chosen for our study. The professional online questionnaire platform (www.wjx.cn) facilitated feedback on from geographically diverse panelists (Sirevag et al., 2021). This platform, widely used in China, offers online data collection, analysis, and management functionalities, streamlining the survey process compared to traditional methods like offline or email surveys. Given the prevalence of modern communication technology in China, this approach proved more convenient for researchers. This study acknowledges several limitations. Firstly, relying on expert opinion inherently introduces potential subjectivity. Secondly, the study may not comprehensively cover all relevant topics and exploring the distinction between in-hospital and out-of-hospital MRPs could further enhance understanding. Finally, as the proposed system has not been implemented in practice yet, its real-world application might necessitate adjustments based on actual circumstances.

### 4.4 Further work

This study reached a consensus on the formulation of an evaluation system for rational drug use in the context of Zhejiang Province, serving as a foundation for developing a rational drug use management platform for CKD. This system is specifically designed to assess the implementation of such practices in stage 3-4 CKD patients, providing personalized recommendations that ensure drug use is both safe and effective. Further, with the continuous advancement of technology and the increasing medical needs, it may provides an important reference for rational drug use in patients with other fields, such as cardiovascular and cerebrovascular diseases. It will facilitate doctors in gaining a deeper understanding of patients’ needs and enable them to provide more personalized medical services, thereby fostering the advancement of intelligent healthcare. In the implementation stage, the key to ensure the smooth operation of the system is the accurate clinical judgment of doctors and the comprehensive consideration of patient information. It is imperative to underscore the significance of relying on a comprehensively planned suite of supportive tools and systems. These tools (Reeve, 2020), which facilitate the systematization of medical procedures, possess invaluable application potential for clinicians, rendering them indispensable. They can support physicians in different Settings and meet the care needs of diverse patients. Therefore, we must ensure that these tools are used correctly and implemented efficiently.

## 5 Conclusion

In this study, a consensus was reached for the evaluation system of rational drug use in CKD through two rounds of consultations. The resulting system comprises 20 items categorized into drug selection, dosage assessment, treatment duration, prescription and dispensing practices, patient-related factors and other relevant considerations. This system holds potential for evaluating the rational use of medication in stage 3-4 CKD patients within Zhejiang Province. It further provides a foundation for establishing a rational drug use management platform dedicated to CKD patients. This platform aims to enhance drug monitoring and ultimately reduce the incidence of MRPs in CKD patients. In the medical field, the management of chronic diseases has always been a complex and critical issue. For patients with chronic diseases, their medication characteristics often share some common features, such as the use of multiple drugs and individualized medication. Currently, the concepts of precision medicine and personalized treatment are gradually becoming popular. Against this background, our research results have the potential and value to build a rational drug use platform for other disease areas, which is expected to become a reference model in this field. We look forward to the dissemination and application of this platform could offer benefits to a wider range of patients.

## Data Availability

The original contributions presented in the study are included in the article/supplementary material, further inquiries can be directed to the corresponding authors.
